# Parental Depression Symptoms and Internalizing Mental Health Problems in Autistic Children

**DOI:** 10.1007/s10803-022-05518-x

**Published:** 2022-03-24

**Authors:** Brianna Piro-Gambetti, Jessica Greenlee, Emily J. Hickey, Jennifer M. Putney, Emily Lorang, Sigan L. Hartley

**Affiliations:** 1 Waisman Center, University of Wisconsin-Madison, 1500 Highland Ave, Madison, WI 53705 USA; 2 School of Human Ecology, University of Wisconsin-Madison, Nancy Nicholas Hall, 1300 Linden Ave, Madison, WI 53706 USA; 3 Psychology Department, Kenyon College, Gambier, OH 43022 USA; 4 Room 453, 1500 Highland Ave, Madison, WI 53705 USA

**Keywords:** Autism, Depression, Family, Mental health, Parent, Internalizing

## Abstract

Autistic youth are at risk for internalizing mental health problems such as depression and anxiety. Similarly, parents of autistic youth report higher levels of depression than parents of typically developing children. The goal of this study was to examine bidirectional associations between parent depression symptoms and the internalizing problems of autistic youth in 188 families across four time points (T1–T4; spaced 12 months apart). A cross-lagged panel model revealed that mother (T1 and T2) and father (T1) depression symptoms positively predicted the youth’s internalizing problems 12 months later. The youth’s internalizing problems at T3 positively predicted maternal depression symptoms at T4. Future research should explore genetic and environmental pathways that link parent depression and internalizing problems in autistic youth.

## Introduction

Autism spectrum disorder (ASD) is a neurodevelopmental condition that is estimated to occur in 1 in 54 children in the U.S. (Maenner et al., 2020). Hallmark characteristics of ASD include restricted and repetitive behaviors and difficulties in social communication that create challenges in everyday functioning (American Psychological Association [APA], 2020). Internalizing mental health problems, such as depression and anxiety, are common co-occurring conditions in autistic youth (Kaat & Lecavalier, 2013; Lopata, et al., 2010). It is estimated that 14–20% of autistic youth experience at least one depressive episode by the age of 18 years (Upthegrove et al., 2018), and nearly 40% experience clinically elevated anxiety symptoms or meet criteria for an anxiety disorder (van Steensel et al., 2011). According to the developmental psychopathology framework (e.g., Cicchetti & Howes, 1991; Davies & Cicchetti, 2004; Kerig, 2016), the high risk for depression and anxiety symptoms in autistic youth may be attributed to genetic and neurobiological vulnerabilities as well as ongoing reciprocal transactions between the individual and their environment. The present study sought to understand the bidirectional associations between the depression symptoms of parents and the internalizing mental health problems of autistic youth, as these outcomes may be linked in both genetic and environmental ways.

The developmental psychopathology framework posits that mental health is influenced by a complex interaction of biological, psychological, social, and contextual factors that accumulate and unfold over time (Cummings et al., 2000; Eme, 2017). This framework suggests that autistic youth both actively shape and are shaped by their environment (Masten & Cicchetti, 2010). One salient environmental factor for the mental health of autistic youth is the mental health of parents. Parents of autistic youth have an elevated risk for depression relative to adults in the general population (Cohrs & Leslie, 2017). For example, a recent systematic review and meta-analysis suggested that 31% of parents of autistic youth exhibit clinically-relevant depression symptoms (Schnabel et al., 2020), with individual studies reporting that up to 49% of parents report depression symptoms (Al-Farsi et al., 2016). In part, this elevated rate of depression has been linked to the high parenting stress reported by parents of autistic youth (e.g., Baker et al., 2011; Benson, 2018). Indeed, among parents of autistic youth, those who report higher parenting stress or more child-related challenges (e.g., severity of ASD symptoms or mental health problems) also report more depression symptoms (Benson, 2006). Thus, in part, elevated internalizing mental health problems in autistic youth may affect parents in ways that contribute to their own depression symptoms. However, biological parents of autistic youth have been shown to have an elevated rate of depression prior to having their child (e.g., Hagberg et al., 2018; Vasa et al., 2012; Wiggins et al., 2019). Indeed, parental mood disorders have been found to be associated with about a two-fold elevated risk of having an autistic child (e.g. Jokiranta et al., 2013). These findings suggest a potential genetic connection between lifetime risk of depression and having an autistic child. Moreover, maternal depression at earlier life stages (i.e., prior to becoming a parent) has been linked to having an autistic child with greater emotion dysregulation (Wiggins et al., 2019). This is similar to reports of an increased risk for emotion dysregulation in neurotypical children who have parents with a history of depression (Ghaziuddin & Greden, 1998; Weissman et al., 2006). Thus, the increased risk for depression in parents of autistic children could be driven by a combination of biological (including a genetic predisposition) and environmental (including child-related challenges) mechanisms that interact across time.

A family environment involving parent depression may, in turn, contribute to increased internalizing mental health problems in the autistic child. Neurotypical children with depressed parents are 2–3 times more likely to exhibit symptoms of depression and anxiety when compared to children without depressed parents (e.g., England & Sim, 2009; Gentile & Fusco, 2017; Kamis, 2021; Tirumalaraju et al., 2020; Weissman et al., 2006). Depressed mothers self-report that they are less responsive to their child, express less sensitivity, provide less positive engagement, and are less consistent or constructive in their parenting strategies (Bayer et al., 2006; Goodman et al., 2011; Murray et al., 2003; Rueger et al., 2011), all of which are parenting behaviors that can contribute to depressed and anxious affect in children (Aktar & Bӧgels, 2017; Cummings et al., 2000Goodman & Gotlib, 1999). Similar patterns have been found in parents of autistic children (e.g., McRae et al., 2018; Rodriguez et al., 2019; Zaidman-Zait et al., 2014). For example, in a sample of 150 families of children with ASD, mothers with less (versus more) depression symptoms were warmer and less critical of their autistic child in a five-minute speech sample (Hickey et al., 2020). In a cross-sectional study, McRae and colleagues (2018) found that parental adjustment, based on a combined measure of depression and anxiety, predicted both negative parenting behaviors (e.g., harsh or disengaged parenting practices) and higher child internalizing behavior problems in a sample of 67 parent–child dyads (primarily mothers). Thus, parental depression may lead to parenting behaviors (e.g., less warmth and more disengaged and harsh parenting) that contribute to internalizing mental health problems in autistic youth.

### Current Study

The present study explored the bidirectional associations between parent depression symptoms and internalizing mental health problems in autistic youth in a sample of 188 families (n = 376 parents). Analyses drew from data collected at four time points, each spaced approximately 12 months apart. At Time 1 (T1), the autistic child was aged 5–12 years. At each time point, mothers and fathers separately reported on their own level of depression symptoms and rated their child’s frequency and severity of internalizing mental health problems. Drawing on the developmental psychopathology framework and evidence of both genetic and environmental connections between parent depression and mood dysregulation in autistic youth, the following hypotheses were made: (1) at Time 1, parent depression would be positively associated with child internalizing mental health problems; (2) higher parental depression symptoms would predict increases in internalizing mental health problems in the autistic youth 12 months later, across time points; (3) higher internalizing mental health problems in the autistic youth would predict increased parental depression symptoms 12 months later, across time points. A priori hypotheses about mother-father differences were not made.

## Method

### Participants

Participants in the present study were mothers and fathers of autistic youth who were part of a longitudinal study investigating a variety of family experiences. Inclusion criteria included: (1) parent couple who had an autistic child between the ages of 5–12 years at the time of recruitment; (2) parents were 21 years of age or older; (3) child had an educational or medical diagnosis of ASD and the diagnostic evaluation included the autism diagnostic observation schedule (ADOS-2nd edition; Lord et al., 2012); (4) parents needed to be in a cohabiting couple relationship for 3 years or more; (5) both parents had to agree to participate. Recruitment occurred through research registries, and fliers mailed to schools and ASD clinics, as well as placed in community locations. Between 2013 and 2014, 188 parent couples who met study requirements enrolled in the study. In order to assess the ASD symptom severity of the child, parents completed the social responsiveness scale (SRS-2; Constantino & Gruber, 2012). Five autistic children received an SRS-2 total t-score below or equal to 60; but, after reviewing relevant information such as medical/educational records and ADOS, these children were deemed to meet criteria for ASD, and thus were included in analyses.

For the present study, data was analyzed from the first four time points (T1, T2, T3, T4) of the study. Time points were spaced approximately 12 months apart (*M* = 11.66, *SD* = 3.70). The majority of the autistic youth were male (86%) and the breakdown of ethnicities/races were: White, non-Hispanic (83%); Latino (7%); Asian (4%); Black (3%); Native American (1%); and multiple racial/ethnic groups (3%). Approximately one-third (34%) of autistic youth had an intellectual disability (ID). Autistic children were, on average, 7.90 years old (*SD* = 2.25) at T1. Mothers were on average 38.69 years (*SD* = 5.62), and fathers had a mean age of 40.76 years (*SD* = 6.19). The majority of families were composed of both biological parents (n = 167; 89%). Parent couple relationship length was on average 14.55 years (*SD* = 5.59) at T1. Average household income at T1 was $80,000–$89,999. Table 1 provides demographic information.

**Table 1:** Family sociodemographics

Demographic	*M* (*SD/%*)
Mother (*n* = 188)	
Age in years [*M* (*SD*)]	38.69 (5.62)
Father (*n* = 188)	
Age in years [*M* (*SD*)]	40.76 (6.19)
Parent couple	
Couple relationship length, years [*M* (*SD*)]	14.55 (5.59)
Average household income	$80,000–$89,000
Both biological parents [N (%)]	167 (89)
Biological mom, stepdad [N (%)]	11 (5)
Biological dad, stepmom [N (%)]	5 (3)
Both adoptive parents [N (%)]	5 (3)
Target child (*n* = 188)	
Male [N (%)]	162 (86)
Age in years [*M* (*SD*)]	7.90 (2.25)
Child ethnicity [N (%)]	
White, non-Hispanic	156 (83)
Latino	13 (7)
Asian	8 (4)
Black	6 (3)
Native American	1 (1)
Multiple racial/ethnic groups	5 (3)
ID [N (%)]	65 (34.4)

*M* mean, *SD* standard deviation, *N* sample size, *ID* intellectual disability

### Procedure

The Institutional Review Board at University of Wisconsin-Madison approved the present study. Parents provided informed consent prior to study participation. Mothers and fathers separately completed questionnaires about their own depression symptoms and the autistic youth’s internalizing mental health problems and jointly reported on family demographics at each time point. Parents were paid $50 each for this portion of the study at every time point.

### Measures

#### Family Sociodemographics

Parents reported on family sociodemographics including child age (years) and child intellectual disability status (0 = no ID, 1 = ID), based on the presence of an ID diagnosis or an indication that the child met criteria for ID via IQ testing. Parents also reported on their household income (Scale of 1–14; 1 = $1–$9,999, 14 = $160,000+).

#### Parent Depression Symptoms

Parental depression symptoms were assessed using the Center for Epidemiological Studies-Depression Scale (CES-D; Radloff, 1977). The CES-D consists of 20 items that are summed into a total score. Mothers and fathers separately reported on how often they felt each item during the past week using a 4-point scale (e.g., 0 = rarely or none of the time to 3 = most or all of the time). Example items are: (1) I was bothered by things that don’t usually bother me; (2) I felt that I could not shake off the blues even with help from my family or friends. A CES-D total score of ≥ 16 is indicative of clinically significant depression symptoms (Radloff, 1977). In the current sample, the CES-D had high internal consistency across time in mothers (Chronbach’s α = 0.92–0.93) and fathers (Chronbach’s α = 0.89–0.93). Table 2 provides the means and standard deviations for mother- and father-reported CES-D total scores.

**Table 2:** Mother and father reported means, standard deviations for main variables

Measure	Mother *M* (*SD*)	Father *M* (*SD*)	*t*-value	*df*	*p*-value
Time 1	*n* = 187	*n* = 187			
CES-D Total	13.58 (10.35)	11.41 (8.79)	2.289	184	0.023^*^
CBCL Int. T	63.11 (9.44)	61.98 (9.40)	1.345	187	0.180
Time 2	*n* = 162	*n* = 157			
CES-D Total	14.85 (11.45)	12.59 (9.72)	2.291	154	0.023^*^
CBCL Int. T	61.41 (9.17)	60.68 (9.24)	1.103	156	0.272
Time 3	*n* = 137	*n* = 134			
CES-D Total	14.86 (11.29)	11.14 (9.51)	3.103	130	0.002^**^
CBCL Int. T	61.42 (9.28)	59.82 (8.46)	2.007	131	0.047^*^
Time 4	*n* = 127	*n* = 125			
CES-D Total	14.68 (10.70)	12.40 (10.41)	2.199	116	0.030^*^
CBCL Int. T	61.02 (9.28)	60.48 (9.05)	0.816	121	0.416

*M* mean, *SD* standard deviation, *t*-value value for paired-samples *t*-test, *df* degrees of freedom, *CES-D Total* center for epidemiological studies-depression scale total score (Radloff, 1977), *CBCL Int. T* child behavior checklist internalizing T-score (Achenbach & Rescorla, 2001)

*p < 0.05

**p < 0.01

#### Child Internalizing Mental Health Problems

Child internalizing mental health problems were assessed using the child behavior checklist internalizing subscale (CBCL; Achenbach & Rescorla, 2000, 2001) for ages 1.5–5 years (preschool form) and ages 6–18 years (school age form). The CBCL internalizing scale is composed of 32 items and is broken into three subscales: (1) anxious/depressed; (2) withdrawn/depressed; (3) somatic complaints. Items are scored based on a three-point Likert scale (*0* = *not true, 1* = *somewhat or sometimes true, 2* = *very true or often true)*. In the present study, analyses included the Total Internalizing T-score, which draws on all three Internalizing subscales. The CBCL has been shown to have strong reliability in the ASD population (Pandolfi et al., 2014). In the present sample, the CBCL Internalizing subscale had a high internal consistency across time as reported by both mothers (Cronbach’s α = 0.84–0.85) and fathers (Cronbach’s α = 0.82–85). The means and standard deviations for mother- and father-reported CBCL Total Internalizing T-scores are presented in Table 2.

### Data Analysis Plan

Descriptive statistics and boxplots were used to identify potential outliers and examine the distribution of study variables. In order to examine relatedness among the variables, Pearson correlations between parental depression and child internalizing problems were examined. In addition, to determine covariates to include in primary analyses, Pearson correlations assessing the association between the main study variables (e.g., parental depression, child internalizing mental health problems) and family sociodemographics (e.g., child age, child ID status, and household income) were also conducted.

Structural equation modeling (SEM) was utilized to explore the bidirectional effects between parental depression symptoms and child internalizing mental health problems. Specifically, a multi-group cross-lagged panel model was conducted in MPlus7 (Muthen & Muthen, 2012), using maximum likelihood parameter estimators. Full information maximum likelihood (FIML) was used to account for missing data (Little, 2013). The cross-lagged panel model allows for the examination of bidirectional associations as well as controls for the stability of variables over time (Kearney, 2016). Lagged paths from T1 to T3 as well as from T2 and T4 were added for model stability. Parental depression symptoms and child internalizing mental health problems were entered as continuous variables, and mothers and fathers were entered as a dichotomous grouping variable (e.g., mothers = 1, fathers = 2). Based on recommendations from Little (2013), various model fit indices were examined including the chi-square statistic, root mean squared error of approximation (RMSEA), Tucker–Lewis index (TLI), and comparative fit index (CFI). Models included all 188 T1 families given the theorized genetic (in addition to environmental) pathways of effects between parent depression and child internalizing mental health problems, however, the above SEM models were re-run in follow-up analyses excluding the 21 families with a non-biological parent.

## Results

### Preliminary Analyses

The main study variables (parental depression symptoms and child internalizing mental health problems) were normally distributed without skew (kurtosis range for CES-D = 0.620–1.476; − 0.205 to 0.521 for CBCL). MCAR tests indicated that data were missing completely at random on the main study variables (*X*^2^ = 15.38–29.06, *ps* > 0.05). At T1, 188 families completed the study. Of these families, 61 did not complete one or more of the subsequent time points (completed N: T1 = 188; T2 = 163; T3 = 137; T4 = 127). There were various reasons for study attrition, including no longer being interested in participating, being too busy, and unable to be contacted. Attrition analyses were conducted in order to examine whether families who completed all time points (coded as 0 for “completers”) differed from families who had missing data on one or more time points (coded as 1 for “incompleters”) on the main study variables of parental depression and child internalizing mental health problems. Independent t-tests revealed no significant differences between the two groups in parental depression [mother report: *t*(183) = 0.358, *p* = 0.721; father report: *t*(186) = − 1.102, *p* = 0.309] and child internalizing mental health problems [mother report: *t*(186) = 0.244, *p* = 0.808; father report: *t*(186) = − 0.831, *p* = 0.407]. Additional independent *t* tests also indicated no significant differences between the families who completed all four time points and those who did not in regards to sociodemographics variables.

Means and standard deviations for the main study variables are provided in Table 2. Additionally, within-couple differences between mother and father reports were examined through paired sample *t*-tests, and these values can also be found in Table 2. Mothers and fathers reported significantly different scores on the CES-D at all four time points, with mothers reporting higher levels of depression than fathers. Table 3 displays the number and percentages of mothers and fathers with a CES-D score in the clinical range across the four time points and were as follows: T1 61(33%) of mothers and 46(25%) of fathers; T2 59(36%) of mothers and 47(30%) of fathers; T3 54(39%) of mothers and 29(22%) of fathers; T4 49(39%) of mothers and 29(23%) of fathers. Overall, 33–68 (26–36%) of youth with ASD had clinically elevated (i.e., *t*-score ≥ 70) internalizing scores as rated by mothers and fathers across the four time points (Table 3).

**Table 3:** Number and percent of mothers, fathers, and children exceeding cutoff for either parent depressive symptoms or child internalizing problems

Family member	Time 1 *n*(%)	Time 2 *n*(%)	Time 3 *n*(%)	Time 4 *n*(%)
Mom	61 (33)	59 (36)	54 (39)	49 (39)
Dad	46 (25)	47 (30)	29 (22)	29 (23)
Child	68 (36)	42 (26)	43 (31)	33 (26)
Mom, dad, and child	12 (6)	14 (9)	9 (7)	7 (6)

Cutoff for parent depressive symptoms is a total score ≥ 16 on the center for epidemiological studies-depression scale (CES-D; Radloff, 1977). Cutoff for child internalizing problems is a child behavior checklist (CBCL; Achenbach & Rescorla, 2001) internalizing *t*-score ≥ 70

Pearson correlations between parental depression symptoms, child internalizing mental health problems, and sociodemographic variables (i.e., child ID status, child age, household income) can be found in Table 4. There were significant positive concurrent associations between mother and father reports of parental depression symptoms and child internalizing problems at all four time points. Additionally, mother report of child internalizing problems at T2 was positively associated with child age (*r* = 0.187, *p* = 0.017). Father report of depression symptoms was negatively associated with household income at all time points (T1: *r* = − 0.164, *p* = 0.028; T2: *r* = − 0.283, *p* = 0.000; T3: *r* = − 0.180, *p* = 0.041; T4: *r* = − 0.252, *p* = 0.006). There were no significant associations between child ID status and the main study variables for mother nor father reports. Given these findings, the cross-lagged panel models controlled for child age and household income. Specifically, we regressed parent depression and scores of child internalizing problems on child age and household income at each time point and saved the unstandardized residual scores. These residual scores were then entered into the cross-lagged panel model.

**Table 4:**
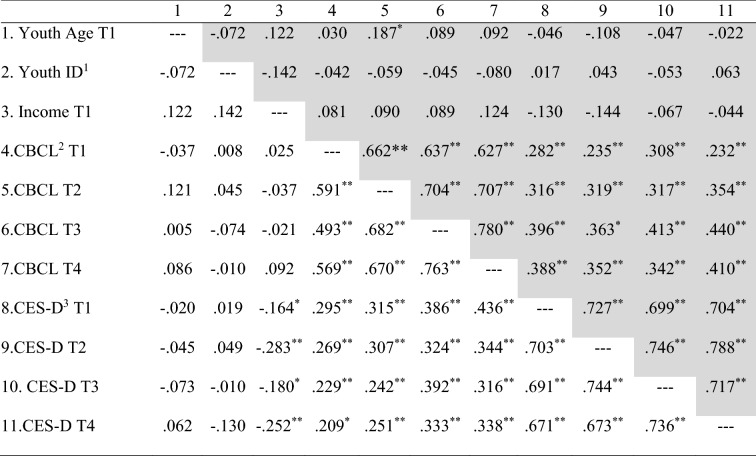
Correlations among study variables for mothers (shaded and above the diagonal) and fathers (unshaded and below the diagonal)

Pearson correlations

*ID* intellectual disability, *CBCL* child behavior checklist internalizing *t*-score (Achenbach & Rescorla, 2001), *CES-D* center for epidemiological studies-depression scale total score (Radloff, 1977)

*p < 0.05

**p < 0.01

### Cross-Lagged Panel Models

**Fig. 1:**
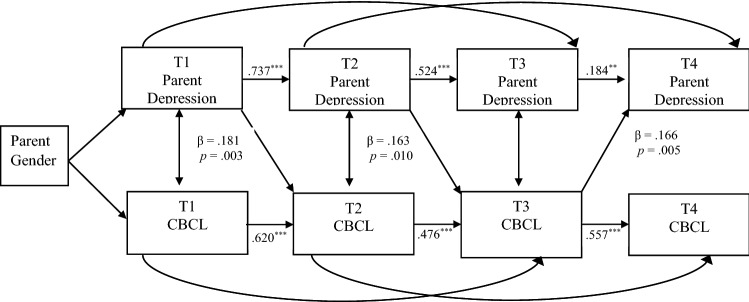
Results of the cross-lagged panel model for mother-reported parent depression and youth internalizing mental health problems, controlling for child age and household income. Values are standardized path estimates. ^*^p < 0.05, ^**^p < 0.01, ^***^p < 0.001

**Fig. 2:**
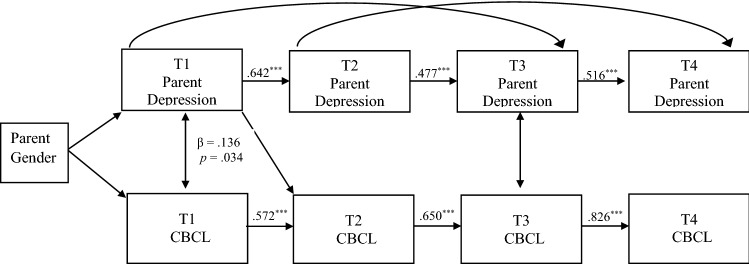
Results of the cross-lagged panel model for father-reported parent depression and youth internalizing mental health problems, controlling for child age and household income. Values are standardized path estimates. ^*^p < 0.05, ^**^p < 0.01, ^***^p < 0.001

Table 5 displays both the standardized and unstandardized path coefficients for the panel model. The model indicated good fit [*X*^2^ (16) = 18.603, *p* = 0.2898; TLI = 0.993; CFI = 0.998; RMSEA = 0.030]. Stability effects in variables across time were present for both mother and father reports. Specifically, mother reports indicated stability in scores of parent depression symptoms (T1–T2: β = 0.737, *p* = 0.000; T2–T3: β = 0.524, *p* = 0.000; T3–T4: β = 0.184, *p* = 0.027) as well as in scores of child internalizing problems (T1–T2: β = 0.620, *p* = 0.000; T2–T3: β = 0.476, *p* = 0.000; T3–T4: β = 0.557, *p* = 0.000). Father reports also indicated stability in scores of parent depression (T1–T2: β = 0.642, *p* = 0.000; T2–T3: β = 0.477, *p* = 0.000; T3–T4: β = 0.516, *p* = 0.000) and in scores of child internalizing problems (T1–T2: β = 0.572, *p* = 0.000; T2–T3: β = 0.650, *p* = 0.000; T3–T4: β = 0.826, *p* = 0.000). After controlling for child age and household income, the cross-lagged panel model revealed that maternal depression symptoms at T1 predicted child internalizing problems at T2 (β = 0.181, *p* = 0.003) and maternal depression symptoms at T2 predicted child internalizing problems at T3 (β = 0.163, *p* = 0.010). In the opposite direction, child internalizing problems at T3 predicted maternal depression symptoms at T4 (β = 0.166, *p* = 0.005). Additionally, father’s depression symptoms at T1 predicted child internalizing problems at T2 (β = 0.136, *p* = 0.034). Figures 1 and 2 depict the significant pathways in the cross-lagged panel model for mothers and fathers, respectively. Given the potential role of genetic mechanisms in linking parent depression to youth internalizing problems, we re-ran the above model after excluding the 21 families that included a non-biological parent(s). All significant pathways remained and thus models with all 188 families are reported on.

**Table 5:** Path coefficients and standard errors for mother and father reports of parent depressive symptoms and youth internalizing mental health problems

Time point	Mother report *β*(*SE*), standardized	Mother report *β*(*SE*), unstandardized	Father report *β*(*SE*), standardized	Father report *β*(*SE)*, unstandardized
Cross effects	Depression → CBCL			
1 → 2	0.181 (0.061)**	0.157 (0.054)**	0.136 (0.064)*	0.145 (0.069)*
2 → 3	0.163 (0.064)**	0.130 (0.051)**	0.061 (0.063)	0.065 (0.067)
3 → 4	0.051 (0.060)	0.041 (0.049)	− 0.008 (0.055)	− 0.011 (0.078)
	CBCL → Depression			
1 → 2	0.032 (0.057)	0.039 (0.070)	0.079 (0.064)	0.078 (0.063)
2 → 3	0.049 (0.058)	0.063 (0.073)	0.004 (0.068)	0.004 (0.068)
3 → 4	0.166 (0.059)**	0.189 (0.068)**	0.116 (0.089)	0.116 (0.090)
Lagged effects				
CBCL				
1 → 3	0.476 (0.077)***	0.243 (0.080)**	0.035 (0.078)	0.036 (0.081)
2 → 4	0.291 (0.073)***	0.300 (0.077)***	0.031 (0.070)	0.044 (0.100)
Depression				
1 → 3	0.281 (0.084)**	0.311 (0.093)**	0.321 (0.090)***	0.344 (0.098)***
2 → 4	0.587 (0.075)***	0.533 (0.074)***	0.212 (0.093)*	0.222 (0.098)*

*CBCL* child behavior checklist (Achenbach & Rescorla, 2001)

*p < 0.05

**p < 0.01

***p < 0.001

## Discussion

The developmental psychopathology framework suggests that mental health problems arise from a dynamic interplay between an individual’s genetic and neurobiological vulnerabilities and an ongoing interplay with the environment (Cummings et al., 2000; Eme, 2017). Within this framework, parental depression may serve as both a genetic and environmental factor that both shapes and is shaped by the internalizing mental health of autistic youth. Consistent with previous reports (e.g., Cohrs & Leslie, 2017; Schnabel et al., 2020), 33–39% of mothers and 22–30% of fathers of autistic youth in the current study reported clinically-elevated depression symptoms, and approximately one-third (26–31%) of autistic youth had clinically-elevated internalizing mental health problems across the three year study. Results also indicate important associations between the internalizing mental health problems of autistic youth and the depression symptoms of parents across time.

There was evidence to suggest that parental depression may contribute to greater child internalizing mental health problems. Specifically, from T1 to T2 (using both mother and father report) and from T2 to T3 (using mother report), higher levels of parent depression symptoms predicted increased internalizing mental health problems in the autistic youth 12 months later. It is important to note that this association could be driven by genes, the environment, or possibly both, consistent with prior work in non-ASD populations (e.g., England & Sim, 2009; Kamis, 2021; Taraban & Shaw, 2018; Tirumalaraju et al., 2020). In the other direction, from T3 to T4, mother-report of child internalizing mental health problems predicted increased maternal depression symptoms 12 months later. Thus, parenting an autistic youth with high internalizing mental health problems may be taxing and contribute to the risk for maternal depression. It is important to note that this direction of effects was present from T3 to T4, but not for earlier time points. Early adolescence is well known as a period of increased risk for internalizing symptoms in neurotypical children, with the incidence of depression doubling from childhood to adolescence (McLaughlin & King, 2014) with a change point around 12 years old (Cohen et al., 2018). Similar patterns have also been found in autistic samples (e.g., Gotham et al., 2015), with evidence of a slightly earlier change point for the increase in internalizing mental symptoms in ASD as compared to neurotypical samples (e.g., Schwartzman & Corbett, 2020). In the present study, at T3 autistic youth were aged 8–15 years (*M* = 10 years). It is also possible that internalizing mental health problems may present in different ways across development and/or it may be more difficult for mothers to manage these problems in older autistic children and autistic adolescents as compared to in younger autistic children. Moreover, overtime, the internalizing mental health problems of the autistic youth may take a greater toll on mothers as they are increasingly seen as chronic problems.

The connections between parent depression and child internalizing problems were most pronounced for mothers. It is unclear why we only found one effect for father report (e.g., father depression at T1 predicted child internalizing at T2). Some research has found that mothers report higher levels of youth internalizing mental health problems, suggesting that mothers could be more aware of these problems and/or observe more of them than fathers, albeit findings are mixed (e.g., van der Veen-Mulders et al., 2017). In part, this could be related to findings that mothers tend to report taking on more parenting responsibilities, on average, than fathers in families of autistic youth (e.g., Hartley et al., 2014; May et al., 2017). As a result, maternal depression symptoms may be more strongly influenced by and be a stronger influencer of internalizing mental health problems in autistic youth than paternal depression symptoms. However, it is important to note that mothers reported more depressive symptoms than fathers, on average. The higher level of depressive symptoms may have biased mothers’ rating of the son/daughter/s internal mental health problems to be more in line with mother’s own mood. In addition to environmental pathways, it is possible that genetic pathways linking parental depression symptoms and the internalizing mental health problems of autistic youth are stronger in mothers than fathers. Indeed, in previous epidemiological research studies showing a link between depression at earlier life stages and later risk of having an autistic child, effects were stronger in mothers than fathers (Cohen & Tsiouris, 2006; Goodman & Gotlib, 1999; Tirumalaraju et al., 2020). More research is needed to explore these and other potential mechanisms.

### Study Strengths, Limitations, and Future Directions

The present study had many strengths including the cross-lagged panel modeling approach which allowed for the examination of bidirectional pathways between parent depression symptoms and child internalizing mental health problems across time in both mothers and fathers of autistic youth. That said, our sample primarily consisted of White, non-Hispanic parents, which limits the generalizability of findings. Future research is needed to determine whether the pattern of effects would remain in larger and more diverse samples. Our sample also only included coupled parents; thus, findings may not be applicable to the experiences of families in single-parent households. Although attrition analyses revealed no significant differences at T1 between families who did and did not complete all time points, families may have differed in other areas not examined. The present study also focused on a single reporter (e.g., mother report of parent depression predicting mother report of child internalizing mental health problems) because we wanted to examine perspectives unique to each parent. In other words, each parent has experiences within their parent–child relationship separate from the other parent’s experiences. Given what we know of the effects of parental mental health on perceptions of child behavior (e.g., Gartstein et al., 2009), we acknowledge that this may have inflated associations. Nevertheless, we believe the single-reporter method is important to understand as the transactional nature of the parent–child relationship may perpetuate both parental depression as well as child internalizing symptoms. It is also important to note that 11% of our families were composed of at least one non-biological parent. These families were removed in follow-up analyses and significant pathways of effects did not change. However, future research should leverage larger samples of non-biological parents to isolate the effects of environmental, relative to genetic, pathways between parental depression and internalizing mental health problems in autistic youth. The present study is also limited in that it did not assess lifetime parent mental health history in biological parents; however, this will also be important for determining genetic pathways of effects. Future research should also examine links between the broader autism phenotype (BAP), which involves sub-clinical autism traits and has an increased prevalence in genetic relatives of autistic youth (Piven et al., 1997). BAP has been posited to overlap to some degree with depression (e.g., Asano et al., 2014; Ingersoll & Hambrick, 2011; Pruitt et al., 2018), and thus parent BAP may also have genetic links to internalizing mental health problems in autistic youth.

### Study Implications

Findings emphasize a need for interventions focused on improving the mental health of both parents and autistic youth. Specifically, it is important to screen for both parent and child mental health conditions in families of autistic youth. It may also be important to offer family-wide, not just individual-level, supports and interventions given evidence that the mental health of parents affects the autistic youth and vice versa. As a result, the mental health of both the parent and autistic youth may need to be targeted for optimal gains. Indeed, there is evidence from non-ASD populations that if parents are depressed, treatment-related gains for the children are reduced (e.g., Eckshtain, et al., 2019). Examples of family-wide programs could include mindfulness training and psychoeducation on child emotion and behavior management for parents combined with cognitive behavioral therapy for the autistic youth. Future research should explore the mechanisms that underlie the potentially genetic and environmental pathways (and specific parenting and child behaviors that may mediate the environmental effects) between parent depression and internalizing mental health problems in autistic youth.
